# Primary Motor Cortex Activation during Action Observation of Tasks at Different Video Speeds Is Dependent on Movement Task and Muscle Properties

**DOI:** 10.3389/fnhum.2017.00010

**Published:** 2017-01-20

**Authors:** Takefumi Moriuchi, Daiki Matsuda, Jirou Nakamura, Takashi Matsuo, Akira Nakashima, Keita Nishi, Kengo Fujiwara, Naoki Iso, Hideyuki Nakane, Toshio Higashi

**Affiliations:** ^1^Department of Community-based Rehabilitation Sciences, Unit of Rehabilitation Sciences, Nagasaki University Graduate School of Biomedical SciencesNagasaki, Japan; ^2^Research Fellow of the Japan Society for the Promotion of ScienceTokyo, Japan; ^3^Department of Occupational Therapy, Unit of Physical and Occupational Therapy, Nagasaki University Graduate School of Biomedical Sciences Health SciencesNagasaki, Japan; ^4^Department of Macroscopic Anatomy, Nagasaki University Graduate School of Biomedical SciencesNagasaki, Japan; ^5^Department of Psychiatric Rehabilitation Sciences, Unit of Rehabilitation Sciences, Nagasaki University Graduate School of Biomedical SciencesNagasaki, Japan

**Keywords:** action observation, primary motor cortex, motor-evoked potentials, transcranial magnetic stimulation, mirror neuron system, video speed, slow motion

## Abstract

The aim of the present study was to investigate how the video speed of observed action affects the excitability of the primary motor cortex (M1), as assessed by the size of motor-evoked potentials (MEPs) induced by transcranial magnetic stimulation (TMS). Twelve healthy subjects observed a video clip of a person catching a ball (Experiment 1: rapid movement) and another 12 healthy subjects observed a video clip of a person reaching to lift a ball (Experiment 2: slow movement task). We played each video at three different speeds (slow, normal and fast). The stimulus was given at two points of timing in each experiment. These stimulus points were locked to specific frames of the video rather than occurring at specific absolute times, for ease of comparison across different speeds. We recorded MEPs from the first dorsal interosseous muscle (FDI) and abductor digiti minimi muscle (ADM) of the right hand. MEPs were significantly different for different video speeds only in the rapid movement task. MEPs for the rapid movement task were higher when subjects observed an action played at slow speed than normal or fast speed condition. There was no significant change for the slow movement task. Video speed was effective only in the ADM. Moreover, MEPs in the ADM were significantly higher than in the FDI in a rapid movement task under the slow speed condition. Our findings suggest that the M1 becomes more excitable when subjects observe the video clip at the slow speed in a rapid movement, because they could recognize the elements of movement in others. Our results suggest the effects of manipulating the speed of the viewed task on the excitability of the M1 during passive observation differ depending on the type of movement task observed. It is likely that rehabilitation in the clinical setting will be more efficient if the video speed is changed to match the task’s characteristics.

## Introduction

Recent years have seen great advances in brain imaging technology, enabling many researchers to elucidate brain mechanisms that were formerly “black boxes”. Researchers have discovered that the mirror-neuron system discharges not only when an individual performs a specific action, but also while observing others’ actions in a macaque brain (di Pellegrino et al., 1992; Gallese et al., 1996; Rizzolatti et al., 1996; Rizzolatti and Craighero, 2004). In subsequent research, researchers found that similar mirror systems also existed in humans (action observation network; AON). They discovered that observing actions activates the same or related cortical motor areas that are involved in the performance of the actions (Fadiga et al., 1995; Grafton et al., 1996; Buccino et al., 2001; Rizzolatti and Craighero, 2004; Iacoboni and Mazziotta, 2007; Gatti et al., 2016).

The AON is activated both when an action is performed and when the same action is observed being performed by others. This neural system is the basis of action recognition (Gallese et al., 1996; Umiltà et al., 2001; Buccino et al., 2004; Sartori and Castiello, 2013; Naish et al., 2016), action understanding (Hari et al., 1998; Rizzolatti et al., 2001; Kilner, 2011; Jacquet et al., 2016), and automatic imitation (Nishitani and Hari, 2000; Iacoboni, 2005), and it is involved in motor learning (Mattar and Gribble, 2005; Stefan et al., 2005; Lago-Rodriguez et al., 2013).

Therefore, action observation shows promise for application as a potential tool for neuro-rehabilitation (Ertelt et al., 2007; Sugg et al., 2015; Jaywant et al., 2016). Regarding the application of action observation in the field of rehabilitation and sports, it is important to observe the elements of the movement accurately during the process of linking the information obtained from observing others to one’s own motor learning. However, there are doubts as to whether humans are able to observe the detailed elements of quick and complex movements such as a figure skater’s performance. For example, it is difficult for us to understand the detailed elements of rapid movements such as performing a triple axel when watching someone perform them; however, it is easy to understand the elements when watching them in slow motion. Human observational ability is limited, and we cannot register the details of quick and complex movements. When we observe rapid movements, we are able to recognize the elements of the movement more accurately when viewing a video in slow motion rather than at the normal speed. Moreover, viewing slow motion video may enable understanding of strategies to achieve proficiency in a given physical activity. As a result, it is possible that the effects of motor learning through observation are more effective at slow, compared to normal, speeds. In contrast, previous studies have found, using fMRI, that the ventral premotor cortex (PMv) and inferior parietal lobule (IPL) that are the parts of the AON are particularly involved in the subject’s ability to understand motor-related components of observed actions, and that AON activation is dynamically modulated depending on whether the element of movement is recognized or not (Ogawa and Inui, 2012). For these reasons, modulation of the excitability of AON has strong potential to account for different results related to the speed of observed video.

Transcranial magnetic stimulation (TMS) in previous action observation studies has shown that the excitability of the primary motor cortex (M1) is enhanced in the area corresponding to the muscle involved in the movement (i.e., it is muscle-specific) during action observation compared to the baseline condition (Fadiga et al., 1995, 2005; Maeda et al., 2002). This is explained by the assumption that the PMv, an important node in the AON that has strong connections to M1, exerts an influence on M1 activity during action observation. There is some evidence to support this explanation. First, M1 is not modulated during action observation after 15 min of 1 Hz inhibitory repetitive TMS delivered over the PMv (Avenanti et al., 2007). Second, using the bifocal TMS/paired-pulse TMS method to deliver a conditioning pulse over left PMv before delivering a test pulse over left M1 provides the opportunity to investigate the interactions between PMv and M1. The bifocal TMS/paired-pulse TMS studies revealed that PMv-M1 connectivity was enhanced during the action observation; therefore, activity in the PMv facilitates the excitability of M1 during action observation (Koch et al., 2010; Lago et al., 2010). PMv is the key area of the AON that modulates the excitability of M1 during action observation.

However, many details about the modulation of M1 excitability during action observation require clarification. In order to contribute to the development of the utility of action observation in rehabilitation and sports, we have previously investigated how the speed of observed action affects the excitability of M1, as assessed by the size of motor-evoked potentials (MEPs) induced by TMS. We discovered that the excitability of M1 during observation of rapid movement was facilitated under slow speed conditions (Moriuchi et al., 2014). In contrast, a recent study investigated the effect of manipulating the video speed on M1 excitability during the observation of an arm crank exercise (Wrightson et al., 2016). This research revealed no differences in the excitability of M1 under different video speeds. There have been few studies of the effect of video speed on the excitability of M1 during observed actions by others, and many points remain unclear. In our previous study, we investigated M1 excitability during action observation solely at normal vs. slower-than-normal speeds. In contrast, Wrightson’s study also investigated high-speed video, which is interesting from a time-efficiency perspective. In the rehabilitation field, the actions needing remediation are not only relatively rapid movements such as “catching a ball” but also relatively slow movements performed in daily life such as “reaching movements”. Therefore, in order to assess the potential clinical use of speed-adjusted action observation, our study should reflect both differing video speeds and different movements. Thus, the present study investigated the effect of three video speeds (fast, normal and slow) for two types of action (rapid and slow movement) on the enhancement of the excitability of M1 by action observation, as assessed by the amplitude of TMS-induced MEPs.

## Materials and Methods

The aim of this study was therefore to investigate the effect of the speed of different types of action on the enhancement of M1 excitability through action observation, as assessed by the amplitude of TMS-induced MEPs.

### Subjects

Twenty-four healthy volunteers (Experiment 1: 9 men and 3 women, mean age 25.7 ± 5.6 years; Experiment 2: 7 men and 5 women, mean age 29.2 ± 5.2 years) were enrolled in this study after providing written, informed consent. All subjects were self-reported right-handers. Baseline characteristics of subjects are shown in Table 1.

**Table 1:** **Baseline characteristics of subjects (mean ± SD)**.

	Experiment 1	Experiment 2
Age	25.7 ± 5.6	29.2 ± 5.2
Sex (Male/Female)	9/3	7/5
Rest MT (%)	46.6 ± 5.4	44.2 ± 6.6
Stimulus intensity (%)	60.0 ± 8.9	58.5 ± 10.3

*MT, Motor Threshold*.

The present study was based on the global guidelines for care in the use of TMS (Rossi et al., 2009). In the first stage of recruitment, all subjects filled out a questionnaire designed to exclude those with contraindications; however, none reported neurological impairment or contraindications to TMS. The study was approved by the local ethics committee at the Nagasaki University Graduate School of Biomedical and Health Sciences. All experimental procedures were conducted in accordance with the Declaration of Helsinki (World Medical Association, 2013).

### Experimental Set-Up

Subjects were seated on a reclining chair 80 cm away from a PC monitor (RDT234WX-Z, MITSUBISHI, Japan, 23-inch, resolution, 1920 × 1080 pixels; refresh frequency 60 Hz) and instructed to keep both hands in a pronated position on a horizontal board attached to the chair’s armrest. They were instructed to keep the right forearm as still and relaxed as possible while paying attention to the visual stimuli presented on the PC monitor. To ensure passive observation of the video clips, the experimenter’s only instruction to the subjects was “You should stay alert while observing a hand,” before starting the experiment.

### Experimental Stimuli

We shot two types of video for the experimental task: one was rapid and the other was slow. The definition of a rapid or slow task was whether or not the elements of the movements could be recognized at a normal playback speed. We shot the task movies with an electrode placed on the actor’s skin and set the timing of the TMS trigger by means of electromyogram (EMG) data during task execution.

#### Experiment 1: Rapid Movement (Catching a Ball)

**Figure 1:**
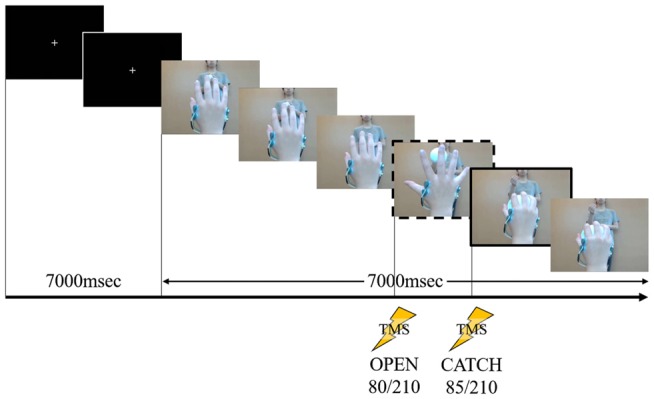
**A sequence of stills from the video clip used in Experiment 1.** The person in the background is the pitcher. The frame in the dashed box is at “open” and the frame in the solid box is at “catch.” During the first 7000 ms, a white cross in the center of a black screen was presented. The first black screen was presented at 7000 ms for all speeds (fast, normal and slow). Following this the action was displayed, the timing of “open” occurring at 80/210 frames after action onset (after the white cross disappeared). After 85/210 frames of the action, the timing of “catch” occurred. This stimulus lasted 14,000 ms at normal speed. Transcranial magnetic stimulation (TMS) stimulation was delivered at one of these two time points in a given showing.

We used the same task in Experiment 1 for rapid movement as in our previous study (Moriuchi et al., 2014). We filmed a model from the first-person perspective performing a one-handed catch using his right hand. An actor in the background threw the ball toward the model. A sequence of stills from the video clip used in Experiment 1 are shown in Figure 1.

#### Experiment 2: Slow Movement (Reaching to Lift)

**Figure 2:**
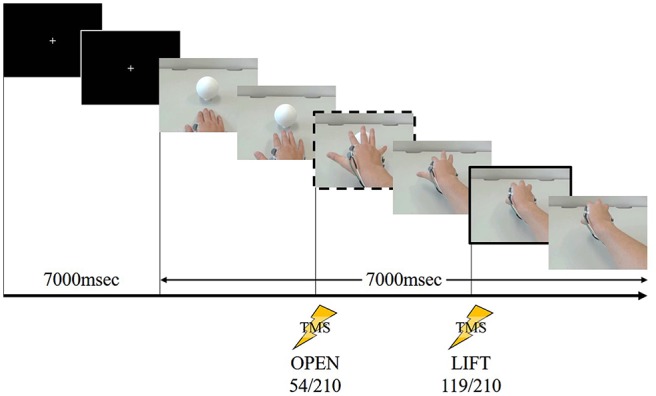
**A sequence of stills from the video clip used in Experiment 2.** The frame in the dashed box is at “open” and the frame in the solid box is at “lift.” During the first 7000 ms, a white cross in the center of a black screen was presented. The first black screen was presented 7000 ms for all speeds (fast, normal and slow). Following this the action was displayed, the timing of “open” occurring at 54/210 frames after the action onset (after the white cross disappeared). After 119/210 frames of the action, the timing of “lift” occurred. This stimulus lasted 14,000 ms at normal speed. TMS stimulation was delivered at one of these two time points in a given showing.

We used the same task as many other TMS studies for the action observation (Alaerts et al., 2012; Tidoni et al., 2013) in Experiment 2. We filmed a model from the first-person perspective performing a one-handed reaching and lifting task using his right hand. A sequence of stills from the video clip used in Experiment 2 are shown in Figure 2.

The ball used in the videos (S3C-NEW, Nagase-Kenko, Tokyo, Japan) is widely used in softball and is the official ball of the Japan Softball Association. The ball was #3 size (perimeter 30.48 ± 0.32 cm; weight 90 ± 5 g; diameter approximately 9.7 cm). The video was recorded using a Web camera (c920r, Logicool, Switzerland) and had a duration of 7000 ms (210 frames). We played the video by presenting a series of single frames, each lasting 33.3 ms, at normal speed (resolution 800 by 600 pixels, color depth 24 bits, frame rate 30 fps), which was sufficiently fast to produce an animation effect.

### Timing of TMS

In order to trigger stimulation at specific times, 210 individual frames were converted to JPEG files and shown in succession to obtain the animation effect. At normal speed, the presentation time of each frame was twice the length of the refresh interval used by the PC monitor (refresh interval = 16.67 ms). The differences in video speed determined the presentation time of each frame (normal speed 33.3 ms/frame; slow speed 66.67 ms/frame; fast speed 16.67 ms/frame). The timing TMS trigger was established for each specific file: it occurred at the same point in the action on the video—for example, when the actors hand was opened fully. Previous studies have found that MEP amplitude correlates significantly with finger aperture during grasping movements and peaks when finger aperture is maximal (Gangitano et al., 2001, 2004). Moreover, previous studies have also revealed a clear correspondence between the time course of the modulation of MEPs during action observation and the temporal pattern of EMG activity (Borroni et al., 2005; Montagna et al., 2005). We decided the timing of the TMS trigger based on these reports. The review written by Naish et al. (2014) showed that “the timing and extent of muscle activity during execution needs to be confirmed by EMG recording, rather than estimated based on knowledge of the primary functions of muscles”. Therefore, we recorded EMG data and assessed the subjects’ muscle activity during task execution.

We used Lab Chart 8 (AD Instruments, Australia) to combine the task movement recordings and the EMG data, and to establish the timing of the TMS trigger. The EMG data were analyzed using the root mean square (RMS) of every 33.3 ms, and the decision on the TMS timing was made by checking the JPEG file and the EMG data as described above. TMS was delivered twice in both experiments. In Experiment 1, we established TMS delivery times as in our previous study (Moriuchi et al., 2014). We decided the trigger point labeled “open” (80/210 frames) was the time the model’s hand had opened to the widest extent just before catching the ball and “catch” (85/210 frames) was the time the model had just caught the ball. By contrast, in Experiment 2, we established the TMS trigger point labeled “open” (54/210 frames) was the time at which the model’s hand aperture was the widest and “lift” (119/210 frames) was the time the model had just lifted the ball.

Prior to the action observation task, M1 baseline excitability at rest was assessed in each subject by recording 20 MEPs while the subject observed a white cross on a black screen under controlled conditions. Subsequently, the experimenter instructed the subject to watch the video without any additional mental effort. All subjects observed the same video clip played at three different speeds (slow, normal and fast). TMS was delivered once for each video clip, randomly at “open” or “catch” (Experiment 1) and “open” or “lift” (Experiment 2). For each subject each speed condition was used in 20 trials. Ten trials used the “open” phase of TMS delivery and ten used the “catch” (Experiment 1) or “lift” (Experiment 2) phase. MEP amplitudes were calculated for each TMS delivery.

We used a computerized pulse-generation system (LabView, National Instruments, Austin, TX, USA). To ensure that TMS was always delivered at the correct time and that the experimental design was correctly implemented, the speed order (slow, normal and fast) was randomized by the experimenter, and the order of TMS delivery times (“open” or “catch”/“open” or “lift”) was randomized by the LabView system. Each of the six possible trial conditions (three speeds × two phases) was replicated 10 times for each subject, for a total of 20 trials per speed, and a further 20 trials were control trials involving TMS and MEP data collection at intervals of 10 s during a sham trial, giving a total of 80 trials per subject.

### TMS and MEP Recordings

Surface EMG activity was recorded in the first dorsal interosseous (FDI) and in the right abductor digiti minimi (ADM), using pairs of 9 mm diameter Ag-AgCl surface cup electrodes (SDC112, GE Healthcare, Japan). Surface EMG signals were amplified and filtered at a bandwidth of 5–3000 Hz using a digital signal processor (Neuropack sigma MEB-5504, Nihon Kohden, Japan), and were transferred to a computer for off-line analysis, using an A/D converter (PowerLab16/30, AD Instruments, Bella Vista, NSW, Australia).

At the beginning of the experiment, we identified the optimal TMS coil position for evoking MEPs in both the right FDI and the right ADM (the hotspot). TMS was delivered to the left M1 hotspot, marked with a pen on a swimming cap covering the scalp of each subject. TMS employed a 70 mm figure-of-eight coil connected to a magnetic stimulator (Magstim 200, Magstim, UK). The coil was placed tangentially to the scalp with its handle pointing backward and rotated approximately 45° away from the mid-sagittal line. Care was taken to maintain the same coil position relative to the scalp throughout the experiment. The resting motor threshold (MT) was defined as the lowest stimulus intensity that evoked an MEP at least 50 μV in amplitude in the right FDI and in the ADM in five out of 10 trials. The test stimulus intensity was set at 110%–130% of the resting MT and produced a control MEP with a success rate that ranged from 35% to 55%, mean 46.6 ± 5.4%, for Experiment 1; 33% to 51%, mean 44.2 ± 6.6% for Experiment 2. The mean size of the control MEP for the FDI and ADM was approximately 0.5–1.0 mV. Throughout the experiments, subjects were instructed to avoid inadvertent movements that could give rise to background EMG activity. For each muscle in each trial, the 20 ms period preceding TMS triggering was checked for background EMG activity.

### Data Analysis

If background EMG data was found, data from both muscles in the trial were rejected. MEP amplitude (peak-to-peak) was measured over each muscle in every trial. MEP amplitude was analyzed using peak-to-peak values and expressed as a percentage of the mean amplitude under control conditions. The data were statistically analyzed using a one-way analysis of variance (ANOVA) with the factor “video speed” (slow, normal and fast) to investigate whether the MEP amplitude was modulated compared with the control condition. Moreover, the data were analyzed statistically using a three-way analysis ANOVA with the factors “video speed” (slow, normal and fast), “muscle” (FDI, ADM), “timing” (open, catch (Experiment 1); open, lift (Experiment 2)). We employed Tukey’s *post hoc* test for multiple comparisons for further analyses. In all analyses, the *p* level for statistical significance was set at *p* < 0.05. All analyses were performed using statistical analysis software (SPSS version 22.0, IBM, Armonk, NY, USA).

## Results

### Experiment 1: Rapid Movement (Catching a Ball)

#### Typical MEP Waveforms

**Figure 3:**
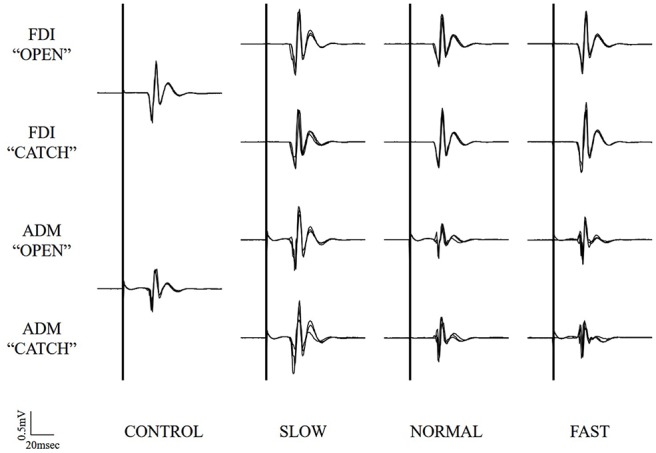
**Typical superimposed waveforms of MEP amplitudes in the three trials at the three different speeds of the right FDI and ADM, recorded from one representative subject in Experiment 1.** MEP, motor-evoked potential; FDI, first dorsal interosseous muscle; ADM, abductor digiti minimi muscle.

Typical superimposed waveforms of MEP amplitudes in the three trials at different speeds of the right FDI and ADM, recorded from one representative subject, are shown in Figure 3. There was a tendency in both muscles for MEP amplitudes to be higher under the observational task than under control conditions. There was also a tendency for MEP amplitudes in both muscles at the timing of “open” and “catch” to be higher under slow-speed conditions than the other speed conditions. In a slow-speed condition, the trend towards increased MEP amplitudes in ADM is higher than the trend in FDI.

#### Mean MEP Amplitude Compared to Control Condition

A one-way ANOVA was performed and a main effect of SPEED was found in the ADM (*F*_(3,33)_ = 7.521, *p* < 0.001, effect size = 0.975, power = 0.406), but not found in the FDI (*F*_(3,33)_ = 0.402, non-significant, effect size = 0.115, power = 0.035). In the ADM, a *post hoc* test revealed that there was no difference between the control condition and the three video speed conditions.

#### Mean MEP Amplitude at Each Speed

**Figure 4:**
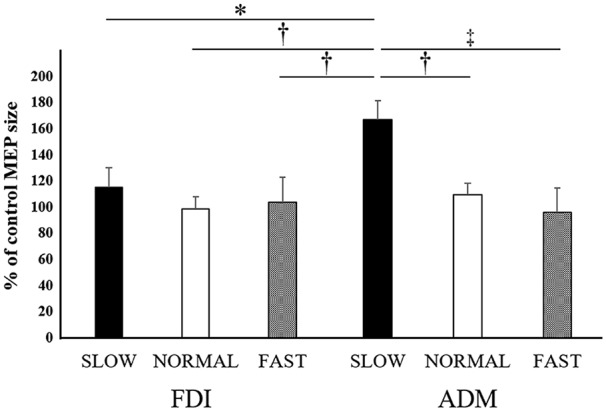
**Mean MEP amplitudes over the right FDI and ADM at the three different speeds in Experiment 1.** Values are expressed as percentage of the control-condition amplitude (*n* = 12). The asterisk (*) represents *p* < 0.05 compared to the “slow speed in ADM”. The dagger-single (†) represents *p* < 0.01 compared to the “slow speed in ADM”. The dagger-double (‡) represents *p* < 0.001 compared to the “slow speed in ADM”.

The mean MEP amplitudes as a percent of control (±SE) induced in the right FDI and ADM in response to single-pulse TMS are shown in Figure 4. Three-way ANOVA was performed and a significant interaction between speed and muscle (*F*_(2,132)_ = 3.417, *p* < 0.05, effect size = 0.049, power = 0.634). To analyze the interaction between speed and muscle, the data were statistically analyzed using a two-way ANOVA with the factors “video speed × muscle” (slow speed in the FDI, normal speed in the FDI, fast speed in the FDI, slow speed in the ADM, normal speed in the ADM, fast speed in the ADM) and “timing” (open, catch). This revealed a significant effect of “video speed × muscle” (*F*_(5,137)_ = 5.134, *p* = 0.0001, effect size = 0.158, power = 0.984). However, “timing” had no significant main effect (*F*_(1,137)_ = 0, non-significant, effect size = 0, power = 0.050). Tukey’s *post hoc* testing detected significant differences in “the slow speed in ADM was 64.5% higher than the normal speed in ADM (95% CI: 11.9%–117.1%, *p* < 0.01), the slow speed in ADM was 79.4% higher than the fast speed in ADM (95% CI: 26.8%–132.0%, *p* < 0.001), the slow speed in ADM was 55.3% higher than the slow speed in FDI (95% CI: 2.7%–107.9%, *p* < 0.05), the slow speed in ADM was 75.2% higher than the normal speed in FDI (95% CI: 22.6%–127.8%, *p* < 0.01), the slow speed in ADM was 67.5% higher than the fast speed in FDI (95% CI: 15.0%–120.1%, *p* < 0.01)”.

### Experiment 2: Slow Movement (Reach to Lift)

#### Typical MEP Waveforms

**Figure 5:**
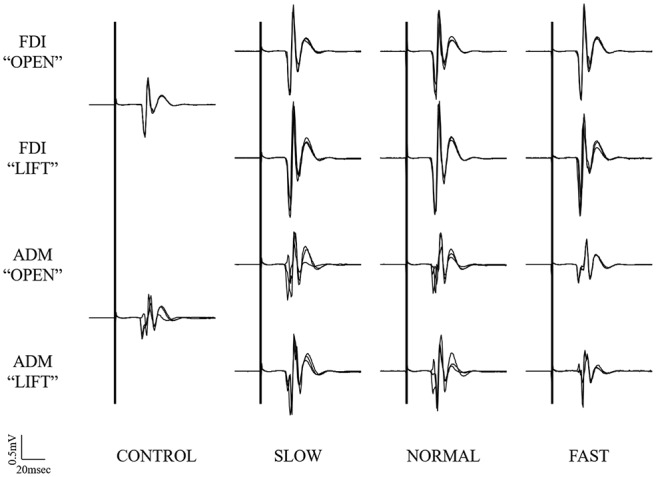
**Typical superimposed waveforms of MEP amplitudes in the three trials at the three different speeds of the right FDI and ADM, recorded from one representative subject in Experiment 2.** MEP, motor-evoked potential; FDI, first dorsal interosseous muscle; ADM, abductor digiti minimi muscle.

Typical superimposed waveforms of MEP amplitudes in the three trials at different speeds of the right FDI and ADM, recorded from one representative subject, are shown in Figure 5. There was a tendency in both muscles for the MEP amplitudes to be higher under the observational task than under control conditions. However, there were no differences for MEP amplitudes in both muscles at the timing of “open” and “catch” among any speed condition.

#### Mean MEP Amplitude Compared to Control Condition

A one-way ANOVA was performed but revealed no significant main effect of SPEED in either the FDI or ADM (FDI: *F*_(3,33)_ = 1.151, non-significant, effect size = 0.095, power = 0.281; ADM: *F*_(3,33)_ = 1.401, non-significant, effect size = 0.113, power = 0.337).

#### Mean MEP Amplitude at Each Speed

**Figure 6:**
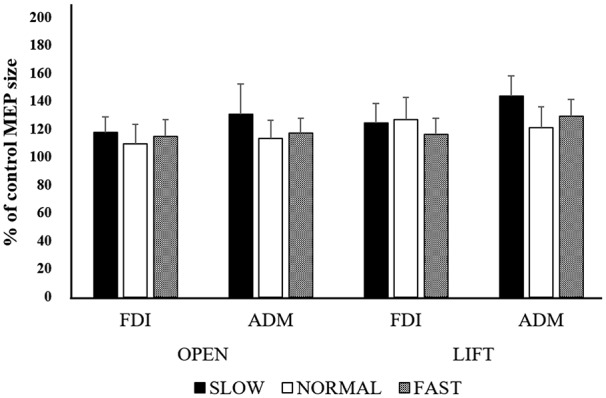
**Mean MEP amplitudes over the right FDI and ADM at the three different speeds in Experiment 2.** Values are expressed as percentage of the control-condition amplitude (*n* = 12).

The mean MEP amplitudes as a percent of control (±SE) induced in the right FDI and ADM in response to single-pulse TMS are shown in Figure 6. Three-way ANOVA was performed and there was no significant main effect for muscle (*F*_(1,139)_ = 0.954, non-significant, effect size = 0.007, power = 0.163), speed (*F*_(2,139)_ = 1.031, non-significant, effect size = 0.015, power = 0.227), or timing (*F*_(1,139)_ = 1.249, non-significant, effect size = 0.009, power = 0.199), nor were there any other significant interactions.

## Discussion

In the present study, we turned our attention to the issue of different types of movement—fast (or rapid) and slow—and investigated the effect of the video speed of the action on the subjects’ action observation; in particular, whether video speed affected the degree of M1 excitation, as measured by the amplitude of TMS-induced MEPs.

### Relationship Between the Type of Observational Task and Video Speed

Some studies have shown that M1 excitability is enhanced by the social features that characterize an observed action (Donne et al., 2011; Bucchioni et al., 2013; Sartori et al., 2013). These studies suggest that M1 excitability is modulated by the participant’s understanding of the social intention and context implied by the observed action. In our study, the experimental action video clearly presented someone catching a ball thrown by someone else in front of him (Experiment 1), or someone reaching and lifting toward a ball (Experiment 2). Therefore, it was possible to understand both the context and intention. These studies would predict that M1 excitability would be enhanced by viewing the video in this context, which indeed occurred. In the current study, we identified that MEP amplitude was found to differ significantly among speeds solely in the rapid movement task. MEP amplitude was not modulated among speeds in the slow movement task. MEP amplitude was higher when subjects observed an action played at a relatively slow speed than at normal or fast speeds. We suggest that these results demonstrate that the effect on M1 excitability of manipulating the speed at which the action is viewed during passive observation differs, depending on the type of movement that is being observed.

Two previous studies of action observation have investigated changes in movement velocity and in the speed at which video of a recorded movement was played. The latter of these, the “video speed study” is most similar to the present study. The former study—on movement velocity itself—identified a significant correlation between M1 excitability and the velocity of the observed upper limb movement: the faster the observed movement, the greater the increase in M1 excitability (Agosta et al., 2016). Further, another “movement velocity study” recording EMG during performing 1.0 Hz to 1.6 Hz flexion extensions of the right wrist revealed that the EMG data of actual movement at 1.6 Hz is significantly higher than at 1.0 Hz (Borroni et al., 2005). The difference in EMG activity might also have affected the modulation of M1 excitability while observing different actual movement velocities.

On the other hand, there are similar “video speed studies” in addition to the present study (Wrightson et al., 2016). In Wrightson’s study, M1 excitability was not modulated between the video speed conditions. The present study also was not modulated on the fast video speed condition in either Experiment 1 and 2. From the results of these “video speed studies”, they revealed that M1 excitability was not modulated during observation of a video playing at a faster than normal speed.

When we compared the movement velocity and video speed studies, we found significant conflicting results between the studies and their characteristics. The “video speed study” found no differences in muscle activity in the video because only the playing speed of the original movie was changed. On the other hand, in the “movement velocity study”, as velocity of movement increased, the amplitude and area of the EMG bursts increased (Mustard and Lee, 1987), therefore, the muscle activity was different among the movement velocity conditions. This difference suggests there might be a different mechanism, rather than just changing movement velocity, that modulates M1.

The present study adopted two types of observational movement task velocity (slow movement and rapid movement). M1 excitability was modulated when observing a rapid movement at slow speed. Moreover, the ADM was significantly facilitated compared to the FDI when a rapid movement was presented at a slow video speed. Everyone expects to be able to recognize elements of rapid movement played at a slow speed that would not be visible to the naked eye at normal or fast speeds. On the other hand, we would not acquire new information such as kinematic movement cues from observing movement at a slow speed. Previous studies using fMRI have found that the PMv and IPL constituent parts of the AON are particularly involved in the subject’s ability to understand motor-related components of observed actions. They suggest that AON activation depends on whether movement elements such as kinematic cues are recognized or not (Ogawa and Inui, 2012). Moreover, the inferior frontal gyrus (IFG), which is strongly connected to M1, reflects the kinematic features of observed actions (Grafton and Hamilton, 2007).

In light of these previous studies, the present study hypothesized that subjects would recognize the elements of the action more easily (and therefore, the AON would be activated) when the replay speed of a video clip of rapid movement was reduced, and the modulation of M1 excitability would be accompanied by AON activation, particularly the PMv and IPL. In contrast, the slow speed movement component of the present study and the arm crank ergometers task of a previous study suggest that the AON was not dependent on the speed of the task video, and that the subject would recognize movement elements without changing the video speed. Therefore, modulation of M1 excitability was not demonstrated under those conditions.

### Difference in Excitability of the Primary Motor Cortex in Muscle Properties

In the present study, video speed modulated M1 excitability only in the ADM but not in the FDI. Previous studies revealed that the muscle-specific modulations of M1 excitability were highly specific to the type of grasping observed. In a whole hand grasp condition (i.e., opposition of the thumb with all fingers), the ADM was facilitated more strongly than the FDI. On the other hand, the FDI was facilitated more strongly than the ADM in a precision grip condition (i.e., the opposition of the thumb with the index finger; Koch et al., 2010; Sartori et al., 2012; de Beukelaar et al., 2016). This visuo-motor transformation involves the anterior intraparietal area (AIP)-PMv-M1 network (Jeannerod et al., 1995). The AIP provides the PMv with the object’s grasp-related information such as its size and shape. Then, PMv outputs the information to the M1 representation of selected muscles involved in the task (Davare et al., 2010). Since the present study adopted the whole hand grasp as an experimental task, the ADM was preferentially activated, in a muscle-specific manner, compared to the FDI. For this reason, the ADM might have been affected by changes in video speed.

An investigation of the effects of changes in the static position of the shoulder on the cortico-spinal excitability of hand muscle revealed that static shoulder position affects the modulation of cortico-spinal excitability only in ADM and not in FDI. This suggests that the FDI is controlled independently without any restrictions of proximal-distal synergies (Dominici et al., 2005). Further, research into the effect of voluntary teeth clenching (VTC), on cortical inhibition revealed that MEPs with posterior-lateral current direction (preferentially elicited I3-wave) in FDI were significantly decreased by VTC when the hand muscle was slightly contracted; however, MEPs in ADM were not altered by VTC. This finding suggests that the FDI muscle may be finely controlled with less restriction of VTC (Takahashi et al., 2006).

In the present study, to summarize, the FDI muscle may be activated independently without being influenced by other factors. This might be the reason why the FDI was not affected by changing video speeds. Our findings showed that there is a possibility that the impact of video speed is dependent on muscle properties. However, since this is still speculative, more studies are needed into the effect of video speed on the FDI and other muscles.

### Limitations and Further Study

The present study did not find that M1 excitability was significantly modulated during action observation compared to a control condition in either experiment condition. However, similar results have been found in previous studies (Sakamoto et al., 2009; Ohno et al., 2011).

A previous study suggested that measurements of corticospinal excitability by TMS during action observation may be an excellent paradigm for probing the AON (Maeda et al., 2002). However, the method used in that study could only probe the AON indirectly, and could not explore the functional roles of other cortical areas in which mirror neurons have been found, such as the PMv and IPL. Several current studies aim to directly explore the individual effects of various neural networks involved in the AON during action observation by means of the bifocal twin-coil TMS method. This is a conditioning-test TMS paradigm in which a test stimulus is applied to M1 after different delays and after a conditioning stimulus is delivered to another cortical area (Davare et al., 2008, 2009; Koch et al., 2010; de Beukelaar et al., 2016). In particular, studies have investigated the excitability of the connections linking PMv and M1 and identified that excitability was not changed at rest, but facilitated in a muscle-specific way during action observation (Koch et al., 2010; de Beukelaar et al., 2016). Activation of the AON during action observation induces specific neurophysiological changes in some of the cortico-cortical connections of the human motor system, such as the PMv-M1. This reinforces the idea that increased excitability of the motor cortex during action observation is mediated by AON activation.

Other methods use transcranial direct current stimulation (tDCS). Subjects underwent cathodal tDCS to the area of the AON, and M1 excitability during action observation was significantly reduced following cathodal stimulation (Enticott et al., 2012). It would be possible to search for AON activation more directly by combining tDCS, adjusted for the excitability of AON, and in the next case assess the excitability of M1 by using the single-pulse TMS method. On the other hand, there is value in using fMRI as a methodology that can search for activation across the entire brain. However, it would be necessary to consider the experimental protocol if fMRI were to be used, because the length of exposure is different for different video playing speeds.

If we wish to discover more about the impact of different video speeds on AON in the future, we will need to research the use of other imaging protocol such as a tDCS and fMRI protocol.

## Conclusions and Clinical Implications

In the present study, we explored the modulation of M1 excitability during two different types of movement with observation of speed characteristics. In conclusion, the effect of video speed was only evident in the rapid movement task under the slow speed condition, and differentially influenced the FDI and ADM. An earlier study had explored similar territory in the past; however, the current study is the first to identify that the type of observational task (slow or rapid) affects the impact of varying video speeds.

Action observation is used in clinical settings where patients watch video sequences depicting activities of daily living, and perform the actions they have observed (action observation therapy). The results of this research, that reveal the impact of different video speeds, and their impact on AON activation will be valuable in clinical settings and, as there are a number of applications for editing movie speed, can relatively easily be made to match video speeds to those optimal for the type of observational movement task required. Further, there is the possibility of changing the video speed to the optimal speed for complex highly skilled movements in the future.

Finally, we believe that the present study’s findings could be relevant to extending the power of action-observation approaches to the rehabilitation of stroke patients.

## Author Contributions

TMo, NI, HN and TH conceived and designed the experiments. TMo, DM, TMa, AN and TH performed the experiments. TMo, KN, KF and TH analyzed the data. JN created the experiment program. TMo and TH wrote the article.

## Funding

This work was supported by a Grant-in-Aid for JSPS KAKENHI Grant Number JP16J05954 to TMo.

## Conflict of Interest Statement

The authors declare that the research was conducted in the absence of any commercial or financial relationships that could be construed as a potential conflict of interest.
